# PhenUMA: a tool for integrating the biomedical relationships among genes and diseases

**DOI:** 10.1186/s12859-014-0375-1

**Published:** 2014-11-25

**Authors:** Rocío Rodríguez-López, Armando Reyes-Palomares, Francisca Sánchez-Jiménez, Miguel Ángel Medina

**Affiliations:** Departamento de Biología Molecular y Bioquímica, Universidad de Málaga, Andalucía Tech, Facultad de Ciencias, and IBIMA (Biomedical Research Institute of Málaga), Málaga, Spain; CIBER de Enfermedades Raras (CIBERER), E-29071 Málaga, Spain

**Keywords:** Functional relationships, Phenotypic relationships, Gene-disease relationships, Systems biology, Network medicine, Network biology

## Abstract

**Background:**

Several types of genetic interactions in humans can be directly or indirectly associated with the causal effects of mutations. These interactions are usually based on their co-associations to biological processes, coexistence in cellular locations, coexpression in cell lines, physical interactions and so on. In addition, pathological processes can present similar phenotypes that have mutations either in the same genomic location or in different genomic regions. Therefore, integrative resources for all of these complex interactions can help us prioritize the relationships between genes and diseases that are most deserving to be studied by researchers and physicians.

**Results:**

PhenUMA is a web application that displays biological networks using information from biomedical and biomolecular data repositories. One of its most innovative features is to combine the benefits of semantic similarity methods with the information taken from databases of genetic diseases and biological interactions. More specifically, this tool is useful in studying novel pathological relationships between functionally related genes, merging diseases into clusters that share specific phenotypes or finding diseases related to reported phenotypes.

**Conclusions:**

This framework builds, analyzes and visualizes networks based on both functional and phenotypic relationships. The integration of this information helps in the discovery of alternative pathological roles of genes, biological functions and diseases. PhenUMA represents an advancement toward the use of new technologies for genomics and personalized medicine.

**Electronic supplementary material:**

The online version of this article (doi:10.1186/s12859-014-0375-1) contains supplementary material, which is available to authorized users.

## Background

Integration of clinical and biomolecular data is a key step in the advancement of current biomedical research and development. One of the greatest limitations of this process is the absence of standard platforms to merge clinical and research studies [1]. Some recent initiatives have focused on data sharing to provide precise phenotypic descriptions of patients in combination with genetic variation [2,3]. An effective integration of clinical features with their molecular context, including genetic, physical and metabolic interactions, is expected to produce new insights for biomedical research [4]. In fact, the phenome and the interactome were recently listed among the five most up-and-coming ‘omes’ that may offer new insights in science [5]. Therefore, new integrative data tools are required to establish these functional and phenotypic links for genome-scale analyses.

Although inherited disorder databases such as OMIM [6] and Orphanet [7], provide extremely valuable details about the molecular nature of pathological conditions, these databases lack direct procedures for integrating biomolecular information. Biomedical ontologies are promising standard resources to address a systematic integration of phenotypes into the molecular background of mutated genomic regions [1,8,9]. For instance, the Human Phenotype Ontology (HPO) currently contains over 10,000 terms that represent each one an individual phenotype [10]. An intuitive approach for determining similarities between sets of ontological terms (HPO terms), that could represent the phenotypic spaces of disorders or even genes, is to estimate their proximity in the ontology.

On the other hand, the Gene Ontology (GO) is an organized vocabulary of terms that can be subdivided into three sub-ontologies: biological processes, cellular components and molecular functions. Genes are associated with consistent annotations that conform sets of GO terms that are useful to describe the cellular and molecular events involving genes [11]. Furthermore, biomolecular interactomes, such as protein-protein interactions and metabolic and gene regulatory networks, should also be used to obtain a systemic view of the molecular and biochemical reactions related to disease-causing genes [12].

In particular, because ontologies have been beneficial in understanding diseases as a set of phenotypes rather than conceptual entities, studying correlations among distinct biological conditions affected by genetic variations would be very useful [13].

The main purpose of this application is to provide a friendly platform that facilitates the analysis of phenotypic and functional information and the discovery of emergent or unnoticed relationships between pairs of genes or genetic diseases. PhenUMA also complies useful biological information from different interactomes, including protein-protein interactions from STRING [14] and metabolic flux correlations [15]. Altogether, PhenUMA may be useful for discovering interesting new insights on or features shared by human diseases, increasing the potential for diagnosis and pharmacological intervention.

## Implementation

### Knowledge base: data processing and storage

**Figure 1:**
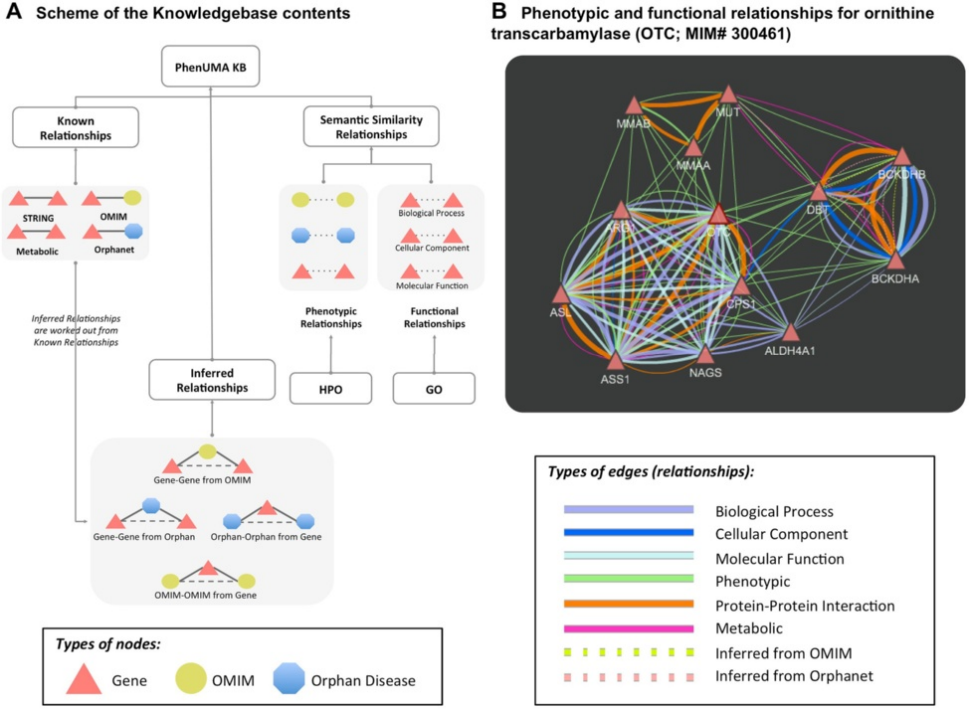
**PhenUMA knowledge base. A**: Schematic representation of the PhenUMA knowledge base contents. Three types of relationships are included in the knowledge base: i) “known relationships” (solid lines) were extracted from the databases OMIM, Orphanet and STRING and also include the metabolic interactions from Veeramani and Bader [15]; ii) “inferred relationships” (dashed lines) were taken from the OMIM and Orphanet known relationships; and iii) “semantic similarity relationships” (dotted lines). For the semantic similarity relationships, scores were calculated using the HPO and GO. Genes (red triangles), OMIM diseases (yellow circles) and Orphanet diseases (blue octagons) are the components of these relationships. This schematic describes how inferred relationships were determined from known relationships; that is, how the dashed lines were deduced from the solid lines. **B**: Illustrative example of integration between phenotypic and functional gene-gene relationships as retrieved in PhenUMA for ornithine transcarbamylase (*OTC*; MIM# 300461) at a medium confidence level.

The initial stages of the development of PhenUMA were focused on building a consistent knowledge base, and subsequent efforts were dedicated to design a user-friendly web application. The knowledge base contains all of the information necessary to create the output networks, and the source data were retrieved from consolidated databases or from inferred relationships determined using different data processing methods (Figure 1A, schematic representation of the knowledge base). The web interface was implemented to make the query execution easier and to allow the visualization of outcome networks according to the Cytoscape Web 1.0.3 utility [16]. The tool was developed in Java, and the database was built using MySQL 5.0.45. PhenUMA and other resources such as tutorials and downloadable processed data are available on the web (http://www.phenuma.uma.es/). An illustrative example of all of the types of gene-gene relationships is shown in Figure 1B.

### Known relationships

The Gene Map file provided by OMIM was used to extract 4,261 relationships between 2,794 OMIM genes and 3,486 OMIM phenotypes; OMIM genes were mapped to their GeneID. The PhenUMA knowledge base also contains the associations between Orphanet diseases and genes. This information was extracted from the file “Diseases with their associated genes”, included at Orphadata [17], and was used to develop 4,472 connections between 2,614 GeneIDs and 2,555 orphan diseases. We also included the diverse interactomes of human protein-protein interactions (96856 relationships) that were found with STRING [14] and 9812 gene pairs that had positive flux correlations in the metabolic network [15].

### Inferred relationships

The inferred relationships between genes or diseases and orphan diseases are due to binary relationships, resulting in four different types of networks. For instance, an inference between two genes will be considered if at least one or more OMIM/Orphan diseases are associated with both genes. A stronger interaction between two genes will be considered when they share more than one disease. Overall, the scores that indicate the intensity of the relationship is the number of disorders involved in the relationship. The same criterion was applied to establish the inferred relationships between OMIM and Orphan disorders. In this case, the number of genes shared by the disorders is considered the score.

### Semantic similarity relationships

HPO and GO were used to calculate the phenotypic similarities between genes or diseases and the functional similarities between genes, respectively. We used Ontologizer 2.0, an open-source tool, to determine the functional similarities, and it was also adapted to compute phenotypic similarities [18]. Each gene or disease is represented by a set of terms that defines its functional or phenotypic profile. Only the most specific terms are included in the annotation files because the “true path rule” is met. This rule implies that each object related to a term also relates to all of the ancestors of this term to the root. For instance, the OMIM (MIM# 200500) disorder “Acheiropody” is associated with both “Humeral hypoplasia” (HP:0005792) and all of its ancestors, such as “Aplasia/Hypoplasia of the humerus” (HP:0006507).

Two different semantic similarity measures that are based on Resnik’s approach were used to calculate the functional similarity among genes and the phenotypic similarity among phenotypic profiles. Both measures are based on the concept of information content (IC), which is calculated using the logarithm of the probability of each term (the ratio of the number of annotations of a term to the total number of annotations). If the probability decreases, then the IC increases, and consequently, the specificity and the informativeness also increase. The semantic similarity between two terms of a given ontology, as proposed by Resnik [19], is determined by the IC of the most informative common ancestor (MICA). The similarity score between groups of terms was obtained by selecting the maximum MICA from all possible pairs of terms. This algorithm has produced suitable results for calculating functional similarity among genes on several occasions [20-22] and is based on the most specific GO terms. This allows relating genes considering the closest molecular mechanisms between them. Regarding to the phenotypic similarity, we have used the complete set of symptoms (HPO terms), associated with a disease or gene, because is more adequate to compare phenotypic profiles. For this reason, we used the method applied by Robinson and co-workers [23], based on Resnik combined with the best-match average. Briefly, if p1 and p2 are two different phenotypic profiles, the semantic similarity of this pair of HPO terms is defined as:

1 $$ sim\left(p1,\kern0.5em p2\right)=\frac{{\displaystyle {\sum}_{ti\in \kern0.5em p1}ma{x}_{tj\in p2}sim}\left(ti,\kern0.5em tj\right)}{\left|p1\right|} $$

where t_i_ and t_j_ represent each HPO term that is included in the profiles p1 and p2. This equation is not symmetric. Robinson and co-workers use a symmetric version for HPO [23]:

2 $$ \mathrm{s}\mathrm{i}{\mathrm{m}}_{symmetric}\left(p1,\kern0.5em p2\right)=\frac{\mathrm{sim}\left(p1,\kern0.5em p2\right)}{2}+\frac{\mathrm{sim}\left(p2,\kern0.5em p1\right)}{2} $$

The annotation files that include the relationships between genes or diseases and their ontological profiles were required to calculate semantic similarity. We downloaded the annotation file “gene_annotations.goa_human”, which relates GO terms to human genes, from the GO website. Two additional files, named “phenotype_annotation.tab” for OMIM and orphan diseases and “gene2phenotype.txt” for gene annotations, were downloaded from the HPO website. In this case, only the annotations of the descendent terms from the “Phenotypic Abnormality (HP:0000118)” term were used for the calculations. This process compiled the associations of 4,965 OMIM diseases plus 3,143 orphan diseases with sets of HPO terms and relationships between 1,806 genes and HPO terms. Table 1 summarizes the different types of semantic similarities processed by PhenUMA.

**Table 1:** **Summary of main relationships in the knowledge base**

**Type of network**	**Type of interaction (source)**	**Nodes**	**Relationships**
***Phenotypic relationships***			
OMIM-OMIM	Inferred by Genes (OMIM)	1843	2885
OMIM-OMIM	Phenotypic Similarity (HPO)	4627	149689^a^
Orphan Disease-Orphan Disease	Inferred by Genes (Orphanet)	1655	3568
Orphan Disease-Orphan Disease	Phenotypic Similarity (HPO)	3068	75924^a^
Gene-Gene	Inferred by OMIM (OMIM)	784	3217
Gene-Gene	Inferred by Orphan Disease (Orphanet)	1641	8292
Gene-Gene	Phenotypic Similarity (HPO)	1681	24902^a^
***Functional relationships***			
Gene-Gene	Functional Similarity (GO Biological Process)	9123	486982^a^
Gene-Gene	Functional Similarity (GO Cellular Component)	6046	565739^a^
Gene-Gene	Functional Similarity (GO Molecular Function)	8087	397683^a^
Gene-Gene	Protein-protein interactions (STRING)	10316	96856
Gene-Gene	Metabolic interactions [Veeramani and Bader[15]]	535	9812

^a^Resulting relationships to apply the respective cutoff for low confidence level.

### Optimal threshold selection of semantic similarities

Each type of semantic similarity calculation requires the establishment of an optimal statistical threshold to differentiate between significant and non-significant similarity scores. Therefore, a minimal meaningful threshold was estimated for each class of phenotypic and functional similarity listed in Table 1. Four different reference datasets were generated from the information in the PhenUMA knowledge base: one for each phenotypic similarity (OMIM-OMIM, Orphan Disease-Orphan Disease and Gene-Gene) and another for all different types of functional similarity. In particular, we compared each dataset of disease pairs, which was inferred from the gene-disease association studies found in OMIM and Orphanet, to the phenotypic similarities between the diseases. The dataset for phenotypic similarities between genes was generated from the union of all inferred pairs obtained from OMIM and Orphanet. The fourth reference dataset resulted from the combination of interactomes from both metabolic and protein-protein interactions; the same dataset was used for all of the functional similarities.

**Figure 2:**
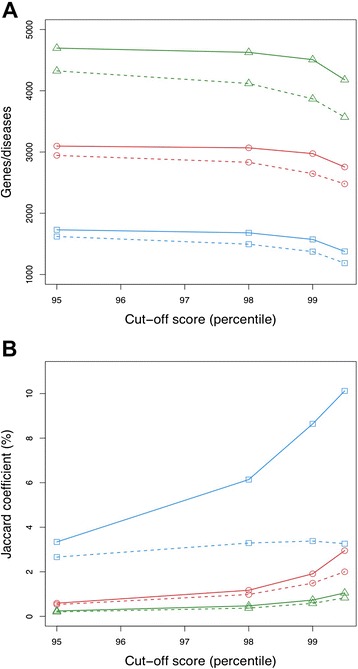
**Effects of phenotypic similarity cutoff variations on the number of elements and Jaccard coefficients.** Computed phenotypic similarities for gene pairs (blue squares), OMIM disease pairs (red circles) and Orphanet disease pairs (green triangles) were filtered at the 95^th^ percentile, and different cutoff scores corresponding to the 95^th^, 98^th^, 99^th^ and 99.5^th^ percentiles were used. The Resnik and Robinson measurements are shown as solid and dashed lines, respectively. **A**: Variations in the number of genes and diseases that are involved in phenotypic similarities at increasing values of the similarity score. **B**: Variations of the Jaccard’s similarity coefficients calculated from the resulting intersection between the phenotypic similarity-based networks and their respective inferred networks is represented as the distinct similarity scores.

Initially, we built a binary classifier system that compares all of the computed scores between semantically similar genes or disease pairs with their respective reference datasets. However, the estimated thresholds in each ROC curve were meaningful (Additional file 1), but they are impractical as optimal cutoffs because of the large size of the resulting networks. Therefore, we analyzed cutoff variations in the phenotypic similarity datasets using a similar approach as in one of our recent studies [13]. First, we removed all pairs of genes or diseases that had a similarity score below the 95^th^ percentile. Next, we studied both the influence of cutoff variations on the number of gene or disease entries and the resulting Jaccard’s similarity coefficients when comparing the semantic similarity networks to their respective reference datasets network (Figure 2). More specifically, the Jaccard’s similarity coefficient represents the number of intersected pairs of gene or disease entries divided by the number of pairs of entries in the union.

As shown in Figure 2A, the number of genes and diseases began to decrease at the 98^th^ percentile of all phenotypic similarities. Robinson’s measurement clearly conserved more genes and diseases at the same cutoff points than Resnik’s did measurement (solid lines above dashed lines, Figure 2A). The phenotypic similarity networks that result in different cutoffs are more similar to the reference dataset networks as we increase the similarity score cutoffs (solid lines above dashed lines, Figure 2B). This trend is especially notable for the evolution of Jaccard’s similarity coefficient for the phenotypic similarity gene networks at the 98^th^ percentile, where Resnik’s measurement has a maximum similarity of approximately 3% and Robinson’s one increases up to 10%. Indeed, this coefficient even decreased in Resnik’s measurement at the 99^th^ percentile (blue squares and dashed line, Figure 2B). The phenotypic similarity disease networks also had slightly higher Jaccard’s similarity coefficients for Robinson’s measurement from the 95^th^ percentile to the top similarity score (red circles and a solid line for OMIM diseases and a green line, Figure 2B).

As it was foreseeable, the semantic similarity measurement applied by Robinson produced better performance for phenotypic similarities than Resnik’s method (see Additional file 1). This analysis revealed the 98th percentile as a suitable threshold that provided a balanced tradeoff between a gain in specificity for phenotypic similarities and a loss of information for disease and gene pairs (Figure 2). For this reason, we selected the 98^th^ percentile of Robinson’s measurement as the lowest similarity value and the minimal appropriate cutoff to build phenotypic similarity based networks.

On the other hand, functional similarities are strongly dependent on large ontological domains that cluster genes with similar scores. Consequently, we set the lower cutoff at the 99.5^th^ percentile, which considerably increases the similarity’s significance and reduces noise from non-informative similarities. Therefore, phenotypic- and functional similarity-based networks were stored in the knowledge base using the 98^th^ and 99.5^th^ percentile as the minimal levels of confidence, respectively (Table 1). All of the scores were normalized following a min-max normalization method, and therefore the scores take values between 0 and 1, where 0 corresponds to the minimal score greater than the cutoff, and 1 represents the highest score for semantic similarity. This method results in confident semantic similarity relationships and a manageable size of networks to be processed by PhenUMA.

## Results

### Network building process

**Figure 3:**
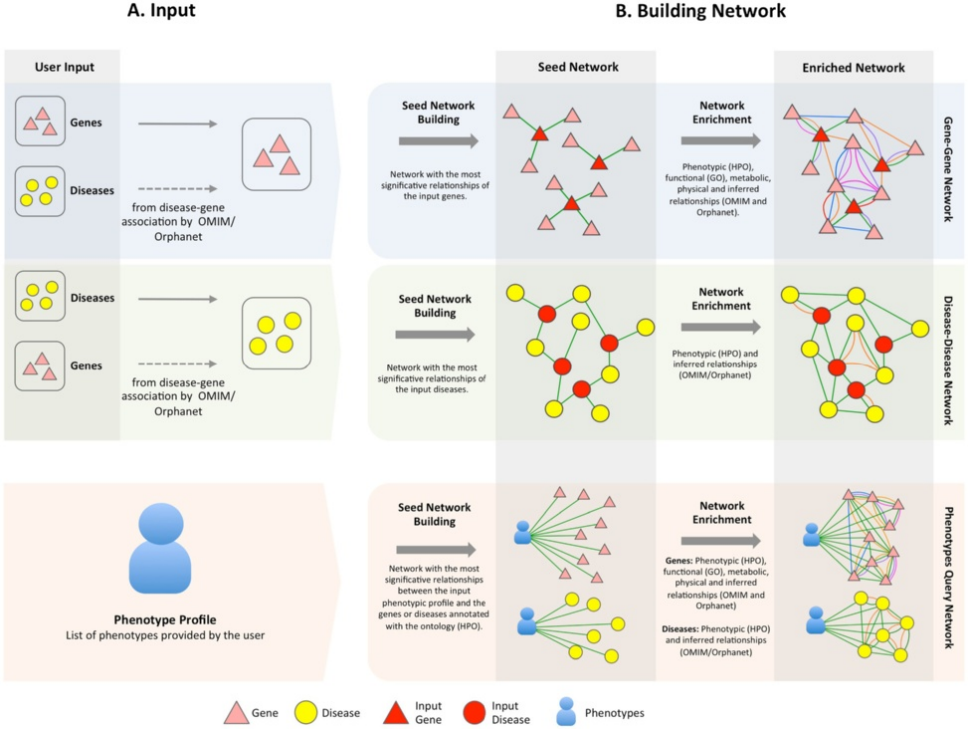
**Building network process. A**: Input provided by the user of PhenUMA. *Gene-Gene network* building allows a set of genes or diseases as input (OMIM or Orphan diseases). In case of providing a disease list, genes associated with each disease (OMIM or Orphanet associations) are use to create the gene-gene network in the building network stage. *Disease-Disease network* can relate OMIM diseases or Orphan diseases and in both cases the input type are similar: a list of diseases or a set of genes. *Phenotype query network* building require of a set of phenotypes (HPO) as input, which is taken as a phenotype profile. **B**: Building network stage is divided in two parts: the seed network building that contains the relationships between de input set (genes, diseases or phenotypes) and the rest of elements included in the database and the network enrichment that consist in the addition of the rest of relationships included in the knowledge base (see Figure 1) between the elements related in each network.

PhenUMA allows the retrieval of information related with a set of genes, diseases or phenotypes of interest. Figure 3 shows the building network stages for each type of input and output. When a query is executed, firstly a seed network is created from the input reported by the user; subsequently, this network is populated with the relationships included in the database for the type of data related (Figure 3B). For example, if a phenotypic similarity network is requested for one gene or one list of genes, the resulting network is populated with the functional, protein-protein interaction, metabolic and inferred relationships (see an example for ornithine transcarbamylase in Figure 1B). PhenUMA allows users to select among three different levels of confidence, termed low, medium and high, for both phenotypic similarities (the 98^th^, 99^th^ and 99.5^th^ percentiles, respectively) and functional similarities (the 99.5^th^, 99.8^th^ and 99.9^th^ percentiles, respectively).

The process of network building is quite different if a set of phenotypes is used as input. In this case, the set of phenotypes is considered as a new phenotypic profile. The similarity between this set and the phenotypic space of other genes or diseases is calculated using Robinson’s semantic similarity measure. In the outcome network, the set of phenotypes is represented as a node, and only the significant relationships (*P-value* <0.05) among the genes or diseases are included. *P-values* are the probability of obtaining a greater score, between the input query and each gene or disease annotated to the ontology, in the comparison with a random set of phenotypes with same size as the input set. The calculation of *P-values* was performed using the Monte Carlo method based on the generation of random samples (1000000 of samples for each size of query from 1 to 10) of phenotypes to calculate a estimation of the probability of a greater score, similar to those used in Phenomizer [24]. For example, if the P-value associated to the score of the relationships between a query of five phenotypes and a disease is 5 · 10^−6^ means that only 5 of 1000000 random combinations of five phenotypes provides a greater score that the input set in the comparison with a specific disease.

### Novel pathological relationships between genes

**Figure 4:**
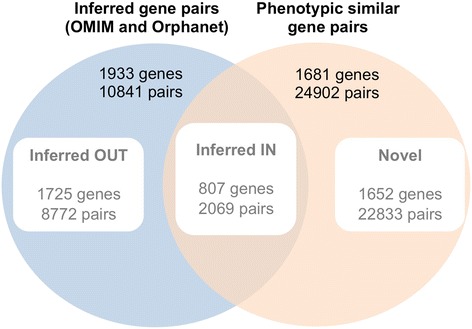
**Subsets of inferred and phenotypically similar gene pairs.** Venn diagram showing the distribution of gene pairs between a dataset of inferred relationships (from the union of OMIM and Orphanet) and the phenotypic similarity gene network at a low level of confidence corresponding to the 98^th^ percentile.

The gene-gene network obtained using semantic similarity methods and the gene-gene inference network from known interactions (both OMIM and Orphanet) were compared to study their mutual coverage. Three distinct subsets were distinguished (Figure 4): inferred pairs of genes that are not included in phenotypic similarity gene network (Inferred OUT), inferred pairs of genes that are in the phenotypic similarity gene network (Inferred IN) and novel pairs of genes that are exclusively in the phenotypic similarity gene network. These latter genes represent more than 90% of all computed phenotypic similarities (22,833 of 24,902 gene pairs). They are considered novel because the involved genes are not co-associated with the same genetic disease based on the current information in OMIM and Orphanet. Notably, 1606 genes in OMIM and 792 genes Orphanet are associated with only one monogenic disease so they would appear as unconnected in inferred networks. Nevertheless, more than 49% and 61% of these genes, respectively, are linked to other genes with phenotypic similarity in PhenUMA.

PhenUMA can detect whether genes are directly or indirectly involved in similar pathological events via the semantic similarity of their phenotypic profiles. For instance, some mutations in *carbonic anhydrase II* (*CA2*; MIM# 611492) are uniquely related to a monogenic disease named osteopetrosis with renal tubular acidosis (MIM# 259730 or ORPHA 2785). When using as output network of gene-gene semantic similarities from HPO with low confidence in PhenUMA, *CA2* shows phenotypic similarities to *TNFSF11* (MIM# 602642), *TBCE* (MIM# 604934) and *SLC4A1* (MIM# 109270). *CA2* also has a physical interaction with *SLC4A1* and a functional similarity for a biological process with *TNFSF11*. In agreement with the whole set of HPO annotations for *CA2*, the most specific clinical features for this gene include: distal renal tubular acidosis (HP:0008341), extramedullary hematopoiesis (HP:0001978), periodic hypokalemic paresis (HP:0008153), optic nerve compression (HP:0007807), elevated serum acid phosphatase (HP:0003148) and diaphyseal sclerosis (HP:0003034). *TNFSF11* presents phenotypic similarities with *CA2* for extramedullary hematopoiesis (HP:0001978), cranial nerve compression (HP:0001293), diaphyseal sclerosis (HP:0003034), hepatosplenomegaly (HP:0001433) and cranial hyperostosis (HP:0004437). Indeed, *TNFSF11* and *CA2* are positive regulators in bone remodeling (GO:0046852) and reabsorption (GO:0045780). *SLC4A1* shares phenotypes with *CA2*, including periodic paralysis (HP:0003768), renal tubular acidosis (HP:0001947) and hypokalemia (HP:0002900) and is also biochemically related to *CA2* by physical interactions. *TBCE* and *CA2* are not functionally associated, but both genes are associated phenotypically with renal tubular dysfunction (HP:0000124) and increased bone mineral density (HP:0011001). This example illustrates the novel phenotypic similarities for *CA2* that are integrated with other functional relationships and additional information processed by PhenUMA. All of these results can be retrieved from PhenUMA combining network visualization, informative panels and other features such as phenotypic and functional enrichment analysis of selected nodes in resulting networks.

### Clustering diseases by phenotypic similarity

**Figure 5:**
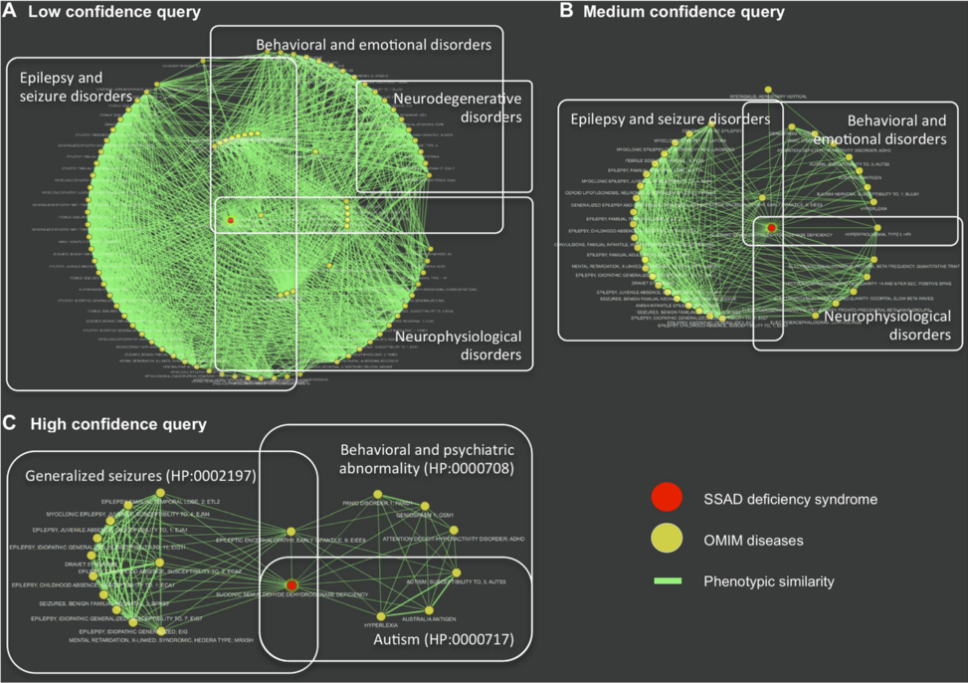
**Phenotypically similar disorders associated with SSADH deficiency at different confidence levels.** PhenUMA results of the query for SSADH deficiency (MIM# 271980) at different levels of confidence **A**: Low, **B**: Medium and **C**: High. All panels are screenshots of the PhenUMA results that were edited to highlight the main clinical features associated with each OMIM disease cluster.

PhenUMA allows users to obtain coherent disease and gene clusters related to a particular disease, gene or set of phenotypes for research purposes. As an example, we will examine succinic semialdehyde dehydrogenase deficiency (SSADHD; MIM# 271980), also known as 4-Hydroxybutyric aciduria, a rare inborn error of metabolism associated with mutations in Locus *ALDH5A1* (*ALDH5A1*; MIM# 610045). We used PhenUMA to search for all of the phenotypic similarities to SSADH deficiency at each of the confidence levels of low, medium and high. These results show how different clusters of diseases are generated and belong to distinguishable groups according to their phenotypic similarity score (Figure 5). For instance, a low cutoff for phenotypic similarity gives four large overlapped and densely interconnected clusters of disorders associated with epilepsy, seizures, neurodegenerative processes, neurophysiological abnormalities and behavioral problems (Figure 5A). SSADH deficiency has a higher frequency of connections to the disorders that involve convulsions, epilepsy or changes in behavior, and the connection becomes more evident when we increase the similarity score to the medium level of significance (Figure 5B). In this case, the established clusters have a more clearly defined structure and relationships to SSADH deficiency. Indeed, three non-overlapped clusters are apparent in Figure 5B. However, although the phenotypic coherence increased, the interconnections between clusters (OMIM diseases) remained abundant in the resulting network (Figure 5B). Therefore, we constrained the query to the most significant phenotypic similarities for SSADH deficiency by selecting the “high confidence” option in PhenUMA.

At least three types of specific phenotypes including behavioral or psychiatric abnormalities (HP:0000708), autism (HP:0000717) and generalized seizures (HP:0002197) involve a succinic semialdehyde dehydrogenase deficiency (Figure 5C). Interestingly, the clusters of disorders associated with behavioral and seizure abnormalities are interconnected by two monogenic diseases: succinic semialdehyde dehydrogenase deficiency (SSADHD, MIM# 271980) and early infantile epileptic encephalopathy-9 (EIEE9, MIM# 300088). Table 2 shows the results of a phenotypic enrichment for the 19 OMIM disorders shown in Figure 5C using the hypergeometric test provided by PhenUMA. These observations demonstrate how phenotypic similarity and network-based methods are useful in studying the pathobiology of human diseases. In particular, this method also provides an alternative procedure to understanding groups of diseases that share similar clinical features.

**Table 2:** **Phenotypic enrichment of SSADHD and high confidence similar disorders**

**HPO term**	**Name**	**Annotated diseases**	**Study**	***P-value***	**MIM diseases**
HP:0002197	Generalized seizures	70	13	4.87E-19	(607628, 607681, 611364, 600669, 608096, 607631, 607208, 300423, 608217, 600131, 271980, 604827, 300088)
HP:0002123	Generalized myoclonic seizures	27	6	2.62E-08	(611364, 600669, 607631, 607208, 271980, 604827)
HP:0002133	Status epilepticus	11	4	3.53E-06	(608096, 607208, 271980, 300088)
HP:0002392	EEG with polyspike wave complexes	4	3	1.35E-05	(607681, 600669, 600131)
HP:0000717	Autism	35	4	5.29E-04	(606053, 238350, 209800, 271980)
HP: 0000708	Behavioural/Psychiatric Abnormality	406	8	4.47E-03	(143465, 606053, 238350, 167870, 209800, 271980, 300088, 190100)
HP:0001311	Neurophysiological abnormality	83	4	1.65E-02	(607681, 600669, 600131, 271980)
HP:0000739	Anxiety	33	3	1.71E-02	(167870, 271980, 190100)

### Comparison with other resources

A comparison between PhenUMA and related web-based tools was performed to analyze several criteria, including the integration of information, the phenotypic information used to relate genes and diseases, the visualization of information and the availability of the datasets. Table 3 summarizes all of the features considered when comparing PhenUMA with other, similar tools.

**Table 3:** **Comparison of PhenUMA with other tools**

**Tool**	**Phenotypic relationships**	**Phenotypic similarity method**	**Gene querying**	**Phenotype querying**	**Information integration**	**Download results**	**Network display**
PhenUMA	Yes	IC-based	Yes	Yes	Yes	Yes	Yes
Phenomizer	Yes	IC-based	No	Yes	No	Yes	No
GeneMania	No	-	Yes	No	Yes	Yes	Yes
PhenomeNET	Yes	Jaccard’s Index	Yes	Yes	Yes	Yes	No
MalaCards	No*	MCRDS	Yes	Yes	Yes	No	Yes

*Mouse Phenotypes (from Mammalian Phenotype Ontology) are related with the disease queried but not Human Phenotypes.

PhenUMA aims to integrate information using network-based methods, and GeneMANIA is a useful example of the integration of biomolecular data [25]. This web interface generates gene networks based on many different types of relationships such as protein and genetic interactions, pathways, coexpression, colocalization and protein domain similarities. However, in addition to functional interactions, PhenUMA also includes the pathological and phenotypic relationships between genes as shown in Table 3. Other tools, such as MalaCards, integrate the pathological and functional information related to human diseases by supplying an extensive repository of different information, where mouse phenotypes are used instead of human phenotypes [26]. Two notable tools that integrate phenotypic information are Phenomizer and PhenomeNET, but both tools are not specifically designed to integrate this information with biomolecular data, which is required for an extensive systemic analysis. Phenomizer demonstrates the potential benefits of semantic- and ontology-based methods when they are applied for the systematic diagnosis of diseases [24]; these features were also included in PhenUMA. PhenomeNET is another tool that allows users to retrieve the semantic similarities between a single OMIM/Orphan disease, gene or phenotype and other genes or diseases, including cross-species information [27] and uses a Jaccard’s index to calculate phenotypic similarity. Conversely, the similarity score between the diseases as calculated by MalaCards, named the Malacards Composite Related Diseases Score (MCRDS), combines an enrichment analysis of disease descriptors with different search engine ranks [26]. The resulting ranked scores in MalacCards are also used to build disease networks based on their shared disease descriptors, but it uses murine phenotypes instead of human phenotypes.

**Figure 6:**
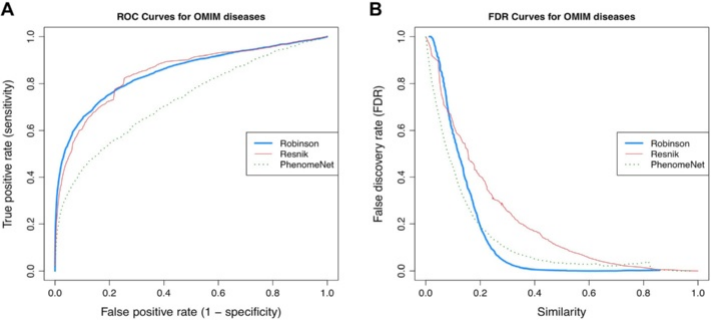
**ROC curve and false discovery rates (FDR) for phenotypic similarities between diseases provided by PhenUMA and PhenomeNET. A**: ROC curves for phenotypic similarities between OMIM diseases. For all the cases we used the same reference dataset. This dataset are all inferred OMIM disease pairs that are those diseases associated with the same gene/s. It is noteworthy that the results from Robinson and Resnik are equivalent to those in Additional file 1: Figure S1A and S1B, respectively, **B**: FDR for increasing values of phenotypic similarity scores.

PhenomeNET and Phenomizer are the most comparable to PhenUMA. Therefore, a more systematic comparison was performed between the results of PhenUMA and PhenomeNET. To do so, we downloaded the file “borderflow-0.1”, which contains relationships and similarity scores between the phenotypes of several species, such as worm, fly, rat, mouse, zebra fish and human, from the PhenomeNET website. Given this cross-species phenotype network, we selected only OMIM disease pairs. A ROC curve was built using the same reference set of inferred relationships between OMIM diseases that share one or several genes. The resulting ROC curves from Resnik’s and Robinson’s measures give better results than those provided by PhenomeNET (Figure 6A). We analyzed the fraction of expected false discoveries by calculating the false discovery rate for each system (Figure 6B). In this case, we observed a lower false discovery rate for PhenUMA, which uses the Robinson’s measure, compared to the similarity score computed using PhenomeNET (Figure 6B). However, PhenomeNET gives a lower fraction of expected false positives than the classical Resnik’s measure.

Finally, using the lists of diseases that are phenotypically similar to SSADH deficiency (OMIM #271980), we made a direct comparison of the results obtained from PhenUMA, Phenomizer and PhenomeNET. First, these lists were ranked by their phenotypic similarity to SSADH deficiency, and the top 10 and 50 of the OMIM diseases were selected. Then, we performed a phenotypic enrichment of each top list using a hypergeometric test and its corresponding Bonferroni correction. In the Table 4, we summarized the results of the phenotype enrichments by comparing them both to the list of phenotypes that are related to SSADH deficiency and to their respective IC values that indicate their level of specificity. For instance, status epilepticus showed the highest IC value, which indicates that it is the most specific phenotype associated with SSADH deficiency (Table 4).

**Table 4:** **Phenotypic enrichment of OMIM diseases similar to SSADH Deficiency (OMIM 271980)**

		**Bonferroni corrected P-values**					
		**PhenUMA**		**Phenomizer**		**PhenomeNET**	
Phenotypes	IC	Top 10	Top 50	Top 10	Top 50	Top 10	Top 50
Status epilepticus	0,709	**7,36E-03**	**1,49E-05**	6,81E-01	5,83E-01	1	1,39E-01
Absence seizures	0,681	**1,21E-02**	**6,75E-11**	**1,02E-02**	**1,91E-11**		2,99E-01
Hyperkinesis	0,658		1			1	1
Hallucinations	0,613		7,55E-01		1		1
Generalized myoclonic seizures	0,604	**6,21E-04**	**2,30E-03**	**5,20E-04**	**6,52E-04**	1	1
Anxiety	0,581	6,90E-02	**6,47E-03**	5,78E-02	4,79E-01		
Autism	0,574	7,76E-02	1,51E-01		5,67E-01		1
Psychosis	0,565	1	**6,61E-04**	1	1		1
Generalized tonic-clonic seizures	0,562	**1,19E-09**	**3,54E-29**	**2,05E-05**	**2,20E-25**	**1,44E-13**	**1,26E-14**
Delayed speech and language development	0,543		1		1	1	1
Aggressive behavior	0,540	1	**6,50E-09**	1	1		1
Hypokinesia	0,491		1				1
EEG abnormality	0,489	1	**4,31E-14**	1	**5,47E-05**	1	1
Increased body weight	0,486		1				
Hyperactivity	0,484		**3,50E-03**		1	1	1
Hyporeflexia	0,437		1			1	1,14E-01
Motor delay	0,420		1		1		1
Ataxia	0,317		1		1	1	**8,27E-21**
Abnormality of eye movement	0,307		8,75E-01			1,54E-01	**1,10E-19**
Muscular hypotonia	0,281		1			1	**2,74E-07**
Intellectual disability	0,214		1		1	8,65E-01	**1,72E-03**
Abnormality of metabolism/homeostasis	0,123	1	1	1	1	1	1

In bold, Bonferroni corrected P-values ≤0.05, hypergeometric tests.

PhenUMA gives a significant enrichment of status epilepticus in the top 10 and 50 of ranked diseases, while no significant enrichment was found for Phenomizer and PhenomeNET. Consequently, the diseases more phenotypically similar to SSADH deficiency are also associated with status epilepticus in PhenUMA. In addition, from the 22 phenotypes annotated for SSADH deficiency, we can count 9 significant phenotypes in the top 50 of the similar diseases retrieved by our system (Table 4). However, Phenomizer and PhenomeNET have only 4 and 5 phenotypes with a *P-value* below 0.05, respectively. Interestingly, there is a gradual enrichment of specific phenotypes in PhenUMA and Phenomizer as we constrain the conditions from the top 50 to the top 10 (Table 4). In contrast, the enrichment of phenotypes in PhenomeNET gives phenotypes with low IC values.

## Discussion

PhenUMA provides an integrative framework for biomedical and biomolecular relationships among genes and genetic diseases by combining network methods and semantic similarity calculations. This integration process uses pathological and functional information from different databases, inferences of already known relationships and computed semantic similarities using biomedical ontologies (HPO and GO), as shown in Table 1. To achieve this goal, PhenUMA uses several biocomputational technologies to unify in the same platform information that apparently is unconnected. One of the primary applications of this platform is to explore how disease-associated genes are phenotypically and functional associated. PhenUMA was shown to be useful for discovering novel pathological relationships between genes and as a new way to study groups of diseases based on the similarity of their phenotypic profiles. These phenotypic similarity relationships are strongly dependent on the ontology structure and the threshold selection. The Human Phenotype Ontology is a standardized platform with recognized clinical value [24], but the selection of an optimal threshold requires reference datasets to assess the precise significance of the similarity score. In PhenUMA, we set a score for semantic similarity that is suitable to detect implicit relationships in databases. The reference datasets used here were built from the inferred relationships (the union of the sets Inferred IN and Inferred OUT of Figure 4) of disease or gene pairs from OMIM or Orphanet that share at least one disease or one gene, respectively. Each type of inference has a different biomedical meaning. For example, an inferred relationship between two disorders, where both present genetic variations associated with the same gene, might indicate a potential functional dependence between these pathologies and the molecular mechanisms involving this gene. If these disorders are phenotypically similar, it supports the hypothesis that perturbations in this gene will produce similar clinical features. Therefore, the resulting thresholds for phenotypically similar diseases are the minimal scores that distinguish disease pairs that are potentially related to the same molecular background. On the other hand, an inferred relationship between genes suggests that both genes could be part of close functional modules. Therefore, mutations in these genes may be canalizing perturbations effects to cause the same clinical features. The resulting optimal threshold is useful for determining the minimal similarity score for two genes that may be involved in the same pathological processes.

Our analysis provides evidence that Robinson’s measurement, which uses the entire phenotypic profile of disorders to calculate similarities between genes and diseases, performs better than the classical Resnik’s measurement (Additional file 1: Figure S1). As the similarity score increases, it implies a higher phenotypic specificity between gene and disease pairs. Robinson’s measure conserves more information (Figure 2A) and the resulting networks are more similar to the used reference datasets (Figure 2B). In addition, PhenUMA provides more confident phenotypic similarities between OMIM diseases than do other similar systems, such as PhenomeNET (Figure 6A and B). To compute similarity scores, both systems use the entire phenotypic profile of OMIM diseases instead of the most specific phenotype in the relationship. It means that the entire phenotypic profile of a disease will be more informative than the most specific phenotype, reinforcing the need for deep phenotyping [1]. Our system also has a lower false positive rate than PhenomeNET (Figure 6B). A possible explanation for these differences is that PhenomeNET uses cross-species information, so it may be influencing the similarity scores.

Furthermore, we also used a case of study of SSADH deficiency to show how phenotypic similarity generates comprehensive clusters of diseases in PhenUMA (Figure 5). The resulting phenotypic enrichments of ranked OMIM diseases by their similarity to SSADH deficiency are quite different for PhenUMA and Phenomizer compared to PhenomeNET. For instance, PhenUMA and Phenomizer, which use the same similarity measures, are more significantly enriched with the clinical features associated with SSADH deficiency than those of PhenomeNET (Table 4). Our results suggest that clusters of phenotypically similar diseases are more coherent in PhenUMA compared to other current similar systems.

Our assessment of the integration of functional and phenotypic relationships was based in a network comparison and correlation analysis of distinct subsets of pairs of genes. In general, phenotypic similarity clusters genes that interact in close molecular and cellular biological conditions. While it remains difficult to systematically distinguish between meaningful relationships and background noise, phenotypic similarity gene network is significantly enriched with functional interactions. For instance, the resulting network of gene pairs from the “Novel subset” is coherent and abundant in functional interactions, especially for protein-protein interactions and functional similarities in biological process (see Additional file 1). In general, protein-protein interactions and pairs of genes with similar cellular localizations likely give more direct evidence for the inferred pathological relationships [28], as observed for the “Inferred IN” and “Inferred OUT” subsets (see Additional file 1). Notably, these results may be influenced by a biomedical research bias, especially for genes that are associated with the same genetic disease [29,30]. Nevertheless, PhenUMA includes the option to filter results with the highest semantic similarity by offering a range of specificity of interactions between genes or diseases. Future improvements on this feature will be needed to extend the validity and the variety of biological interactions.

## Conclusions

In conclusion, the information produced by PhenUMA integrates clinical and biomolecular information to supply wider insights on the phenotypic and molecular characteristics of pathological processes. This tool is useful to help clinical and basic researchers to reinterpret their results and to redesign experiments by considering apparently non-related elements a priori. PhenUMA users can download detailed tutorials and stored networks from the knowledge base on the website. Returns, including comments and criticisms, from final users will be considered for future improvements of this tool.

## Availability and requirements

**Project Name:** PhenUMA**Project home page:**www.phenuma.uma.es**Operating system(s):** platform independent**Programming language:** Java

## Additional file

Additional file 1:
**Evaluation of methods and integration of information.** Evaluation of the measures purposed by Resnik and the approach used by Robinson in the semantic similarity calculation and evaluation of the integration of phenotypic and functional relationships. **Figure S1.** ROC curves for functional and phenotypic relationships. **Figure S2.** Similarity and significance of the intersection between subsets and interactomes. **Figure S3.** Distribution of functional similarity scores in the subsets of inferred and phenotypically similar gene pairs.
