# Supplementary material for: PhenUMA: a tool for integrating the biomedical relationships among genes and diseases
Source: BMC Bioinformatics. 2014 Nov 25;15(1):375. doi: 10.1186/s12859-014-0375-1 (PMC4260198; doi:10.1186/s12859-014-0375-1)
Supplement: Additional file 1: — Evaluation of methods and integration of information. Evaluation of the measures purposed by Resnik and the approach used by Robinson in the semantic similarity calculation and evaluation of the integration of phenotypic and functional relationships. Figure S1. ROC curves for functional and phenotypic relationships. Figure S2. Similarity and significance of the intersection between subsets and interactomes. Figure S3. Distribution of functional similarity scores in the subsets of inferred and phenotypically similar gene pairs. [file 12859_2014_375_MOESM1_ESM.pdf]

Supplementary methods and discussion section for

PhenUMA: A Tool for Integrating the Biomedical Relationships among Genes and Diseases

Rocío Rodríguez-López,[1,2,‡] Armando Reyes-Palomares,[1,2,‡] Francisca Sánchez-Jiménez,[1,2] and Miguel Ángel Medina[1,2,*]

[1]Department of Molecular Biology and Biochemistry, Faculty of Sciences, University of Málaga, E-29071 Málaga, Spain

[2]CIBER de Enfermedades Raras (CIBERER), E-29071 Málaga, Spain

[‡] These two authors contributed equally to this work.

*Correspondence to: Miguel Ángel Medina. Department of Molecular Biology and Biochemistry, Faculty of Sciences, University of Malaga, 29071, Malaga, Spain. E-mail: medina@uma.es

1. Binary classifier system

Four different reference datasets were generated from the information in the PhenUMA knowledge base: one for each phenotypic similarity (OMIM-OMIM, Orphan Disease-Orphan Disease and Gene-Gene) and another for all different types of functional similarity. In particular, we compared each dataset of disease pairs, which was inferred from the gene-disease association studies found in OMIM and Orphanet, to the phenotypic similarities between the diseases. The dataset for phenotypic similarities between genes was generated from the union of all inferred pairs obtained from OMIM and Orphanet. The fourth references dataset resulted from the combination of interactomes from both metabolic and protein-protein interactions; the same dataset was used for all of the functional similarities. All of the gene and disease pairs that were present in both datasets (semantically similar pairs and gene/disease pairs in reference datasets) were considered to be true positives, and the rest were considered to be false positives. Next, we analyzed the performance of this binary classification using a receiver operating characteristic (ROC) curve for each semantic similarity **network**. We considered the scores that maximized the tradeoff between the rates of true and false positives to be thresholds. These scores were determined using Youden's index [1]. In this analysis, we considered Resnik's method and the symmetric version proposed by Robinson [2] for the phenotypic similarities between gene and disease pairs. We calculated functional similarity between genes using only Resnik's method.

The resulting ROC curves show that reference datasets are good estimators for assessing semantic similarities; indeed, each area under the curve (AUC) is above 0.75 (shown in the legend of Additional file 1 Figure S1). All of the thresholds of calculated Youden's indexes (shown in the footnotes of Additional file 1 Figure S1) were meaningful, but they are impractical as optimal cutoffs because of the large size of the resulting networks.

2. Evaluating the integration of functional and phenotypic relationships

The co-dependence of functional and phenotypic relationships was studied for distinct subsets of gene pairs. To do so, we generated five subsets that compare both datasets of inferred and phenotypically similar gene pairs, as suggested by the Venn diagram in

Figure 3 (All Inferred, Inferred IN, Inferred OUT, Novel and All phenotypically similar pairs). However, to evaluate the integration of functional and phenotypic relationships, we used the 99.8^{th} (medium level of significance in PhenUMA) percentile instead of the 99.5^{th} (low level of significance in PhenUMA) percentile to ensure a high confidence in functional interactions and to reduce network sizes to 198,621, 226,854 and 169,626 gene-gene relationships for biological process, cellular components and molecular functions, respectively. This cut-off gives networks for biological processes, cellular components and molecular functions with a similar size compared to other human interactomes (Table 1).

Subsequently, a network comparison between each subset and the functional networks built from biological process similarities, cellular component similarities, molecular function similarities and protein-protein interactions was performed. We excluded metabolic interactions because of their poor representation in some subsets. We used the NeAT Toolbox [3] for this analysis; the similarity between compared networks has been estimated using Jaccard's coefficient (Additional file 1 Figure S2). The statistical significance of the remainder of the network comparisons was calculated using hypergeometric testing (Additional file 1 Figure S2). For this evaluation, phenotypic- and functional similarity-based networks were used at their low and medium level of confidence, respectively. The medium level of significance for functional similarities was selected to reduce noise and network sizes as mentioned above. The resulting functional similarities are highly specific interactions for biological processes, cellular components and molecular functions. In PhenUMA, however, the users have the option to explore functional similarities at the low confidence level.

A Mann-Whitney U test was next used to compare the distributions of functional similarity scores at the intersections of the above network comparisons. The functional similarity scores are greater for all of the pathological subsets of the functional similarity network using the medium level of confidence (Additional file 1 Figure S3A). In addition, we also calculated the average P-value of 1,000 tests using random samples with an equal number of similarity scores as their respective set of intersected scores. These randomizations were generated from the functional similarity networks that were used in the network comparisons for biological processes, cellular components and molecular functions. Average P-values indicate that the functional similarity

distributions in all subsets are significantly greater than those in the entire network (shown in the heat map of Additional file 1 Figure S3B).

Finally, we studied the dependence of functional similarities on phenotypic similarities. To do so, we calculated the Pearson correlation coefficients (PCC) and P-values using a correlation test for the gene pairs at the intersections of the Inferred IN, Novel or All phenotypically similar gene pairs and for each functional network.

3. Results and Discussion

A network comparison analysis of the information integrated in PhenUMA shows a comprehensive correlation between functional and phenotypic interactions. Because the results of theses analysis are somewhat dependent on the threshold of selection, we used a medium level of significance. This threshold implies to generate networks for biological processes, cellular components and molecular functions with a similar size compared to other human interactomes (as those included in Table 1).

Indeed, all of the subsets have the highest values of Jaccard's coefficient for protein-protein interactions compared to the other functional relationships (Additional file 1 Figure S2). Notably, the inferred subset has higher values of Jaccard's coeficient for protein-protein interactions compared to the subset of all phenotypic similarities ("Inferred" and "All pairs", respectively, in Additional file 1 Figure S2). In contrast, the subset of phenotypically similar pairs of genes ("All pairs") exhibits proportionally higher Jaccard's coefficients for functional similarities from the sub-ontology of biological processes (see the purple bars in Additional file 1 Figure S2). These results may indicate the strong support that entails protein-protein interactions in the genes involved in the same or similar pathological processes [4]

However, the distributions of functional similarity scores of all subsets were significantly greater than the corresponding distributions for the total scores of each branch of the Gene Ontology (Additional file 1 Figure S3A and S3B). The "Novel subset" has a lower significance for these differences than the "Inferred IN" and "Inferred OUT" subsets, which have significantly greater similarity scores compared to the whole functional similarity scores (Additional file 1 Figure S3B). The "Inferred OUT" subset is quantitatively lower than "Inferred IN", resulting in phenotypically similar gene pairs likely showing strong functional associations (Additional file 1

Figure S3A). In addition, the distributions of scores for the "Novel" and "Inferred OUT" subsets have a similar behavior for the biological process networks. All of these findings reinforce the hypothesis that novel gene pairs from the "Novel subset", although influenced by unspecific relationships, retain functional coherence.

On the other hand, "Inferred IN", "Novel" and "All phenotypically similar gene pairs" subsets showed significant but weak positive correlations between both the functional similarities of biological processes, cellular components and molecular functions as well as phenotypic similarities (Additional file 1 Table S1). Interestingly, functional and phenotypic score correlations are more positive and significant for the cellular component sub-ontology (Additional file 1 Table S1). This result underscores the biomedical relevance of the coexistence of gene products in the same subcellular location [5].

In summary, phenotypic similarity correlates with the contextual and functional relationships between genes that are based on their co-association with similar biological processes.

4. References

1. Youden WJ: **Index for rating diagnostic tests.** *Cancer* 1950, **3**:32–5.

2. Bauer S, Seelow D, Horn D, Robinson PN, Ko S, Mundlos S: **The Human Phenotype Ontology : A Tool for Annotating and Analyzing Human Hereditary Disease**. 2008:610–615.

3. Brohée S: **Using the NeAT Toolbox to Compare Networks to Networks, Clusters to Clusters, and Network to Clusters**. In *Methods Mol Biol. Volume 804*. Springer New York; 2012:327–342.

4. Goh K-I, Cusick ME, Valle D, Childs B, Vidal M, Barabási A-L: **The human disease network.** *Proc Natl Acad Sci U S A* 2007, **104**:8685–90.

5. Park S, Yang J, Shin Y, Park J, Jang SK, Kim S: **Protein localization as a principal feature of the etiology and comorbidity of genetic diseases**. *Mol Syst Biol* 2011, **7**:1–11.

6. Resnik P: **Using Information Content to Evaluate Semantic Similarity in a Taxonomy**. In *IJCAI*; 1995:448–453.

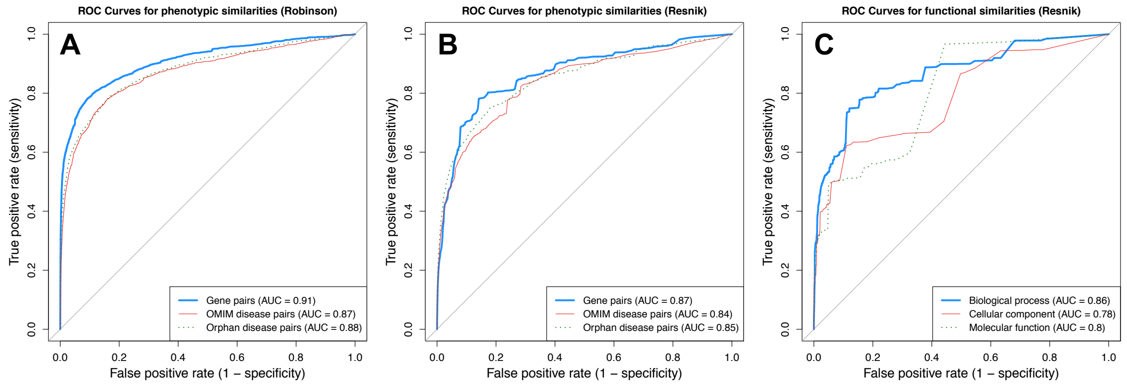

Figure S1. ROC curves for phenotypic and functional similarities. A: Phenotypic similarities were computed using Robinson's method [2]. Youden's indexes represent locations of optimal thresholds in the curve and were calculated for gene pairs (sensitivity, 0.91; 1-specificity, 0.78), OMIM disease pairs (sensitivity, 0.84; 1-specificity, 0.78) and Orphanet disease pairs (sensitivity, 0.84; 1-specificity, 0.78). B: Phenotypic similarities were computed using Resnik's method [6]. Youden's indexes represent locations of optimal thresholds in the curve and were calculated for gene pairs (sensitivity, 0.86; 1-specificity, 0.78), OMIM disease pairs (sensitivity, 0.72; 1-specificity, 0.82) and Orphanet disease pairs (sensitivity, 0.82; 1-specificity, 0.75). C: Functional similarities were computed using Resnik's method [6]. Youden's indexes represent locations of optimal thresholds in the curve and were calculated for biological process pairs (sensitivity, 0.88; 1-specificity, 0.75), cellular component pairs (sensitivity, 0.89; 1-specificity, 0.62) and molecular function pairs (sensitivity, 0.55; 1-specificity, 0.96).

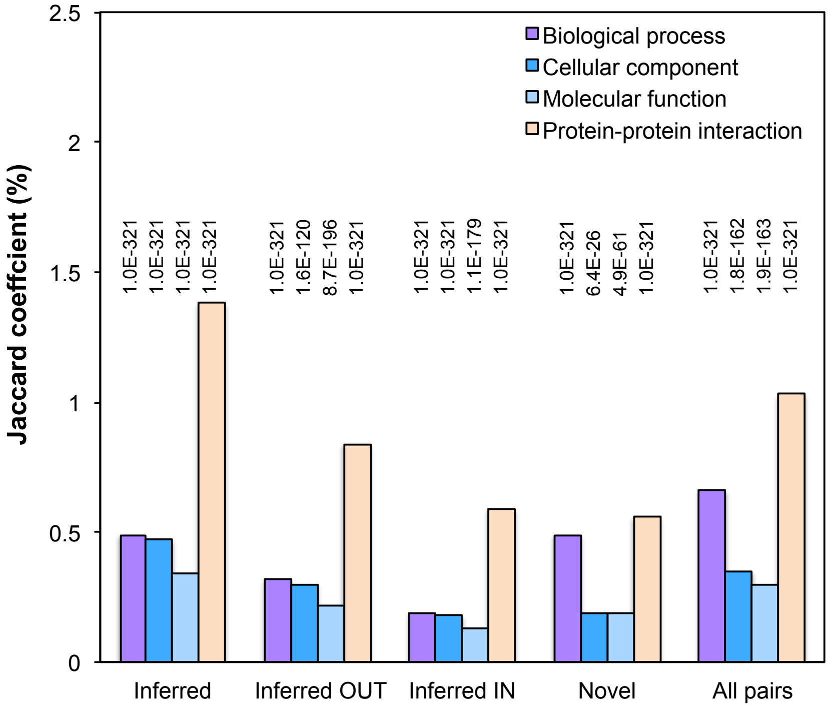

Figure S2. Similarity and significance of the intersection between subsets and interactomes. Bar charts of the resulting Jaccard's similarity coefficients from the network comparison between the functional and phenotypic networks. P-values indicate the significance level of the overlaps from hypergeometric tests. The limit of resolution for this distribution analysis is 1.0E-321.

A

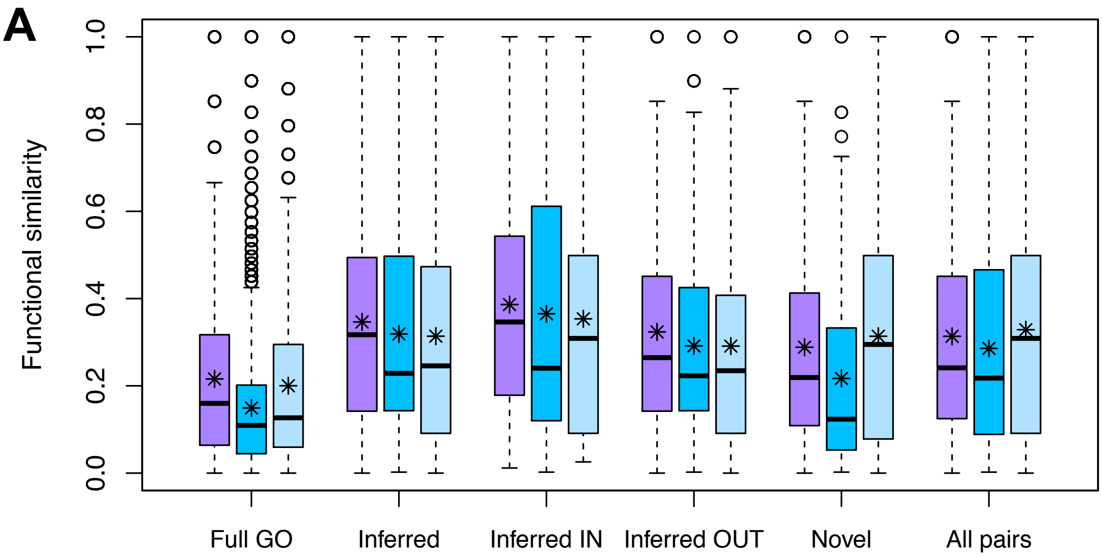

B

Heat map of Mann-Whitney P-values from the distribution comparisons

	**Biological process**	**Cellular component**	**Molecular function**
Inferred	3.53E-30	8.23E-69	3.00E-11
Inferred IN	5.19E-19	2.78E-32	2.32E-06
Inferred OUT	6.00E-13	6.38E-38	3.81E-06
Novel	4.46E-10	1.91E-04	1.37E-06
All pairs	1.87E-22	5.49E-27	1.56E-11

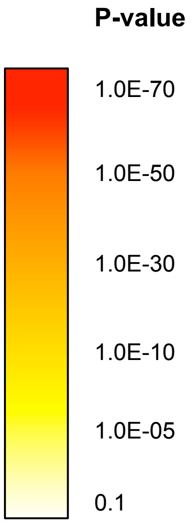

Figure S3. Distribution of functional similarity scores in the subsets of inferred and phenotypically similar gene pairs. A: Box plot of the similarity scores at the intersections between subsets and functional similarity gene networks (biological processes are shown as the purple bars, cellular components as blue bars and molecular functions as light blue bars). "Full GO" represents all similarity scores in the original functional networks filtered at the medium confidence level the 99.8th percentile. B: Heat map plot of the average P-values from the Mann-Whitney tests to compare the similarity score distributions of subsets with random samples of their respective functional similarity gene networks. The significance of this test indicates how distributions are different compared to those in the original networks. The average P-values were calculated using 1,000 tests with different random samples for each case.

Table S1. Pearson correlation analysis between functional and phenotypic similarities.

GO sub-ontology	Inferred IN		Novels		All pairs	
	r (Pearson)	*P-value*	*r* (Pearson)	*P-value*	*r* (Pearson)	*P-value*
Biological process	0.17	2.45E-05	0.12	2.38E-04	0.19	5.28E-14
Cellular component	0.34	0	0.20	4.45E-05	0.34	0
Molecular function	0.29	1.58E-07	0.12	3.08E-02	0.21	8.32E-08
